# Oncolytic Herpes Simplex Virus Therapy: Latest Advances, Core Challenges, and Future Outlook

**DOI:** 10.3390/vaccines13080880

**Published:** 2025-08-20

**Authors:** Yiyang Zheng, Yusheng Pei, Chunyan Dong, Jinghui Liang, Tong Cai, Yuan Zhang, Dejiang Tan, Junzhi Wang, Qing He

**Affiliations:** State Key Laboratory of Drug Regulatory Sciences, National Institutes for Food and Drug Control, Beijing 102629, China; zhengyiyang@nifdc.org.cn (Y.Z.); pysh@nifdc.org.cn (Y.P.); dongchunyan@nifdc.org.cn (C.D.); liangjinghui100@163.com (J.L.); teddyct@163.com (T.C.); zhyuan_cn@126.com (Y.Z.); tandj@nifdc.org.cn (D.T.); wangjz@nifdc.org.cn (J.W.)

**Keywords:** immunotherapy, herpes simplex virus, preclinical and clinical research progress

## Abstract

Oncolytic virus (OV) immunotherapy, particularly with oncolytic herpes simplex virus (oHSV), has become a promising new strategy in cancer treatment. This field has achieved significant clinical milestones, highlighted by the FDA approval of Talimogene laherparepvec (T-VEC) for melanoma in 2015 and the approval of Teserpaturev/G47Δ for malignant glioma in Japan in 2021. This review synthesizes the key preclinical and clinical advancements in oHSV therapy over the last decade, critically analyzing the core challenges in target selection, genetic modification, administration routes, and targeted delivery. Key findings indicate that arming oHSV with immunomodulatory transgenes, such as cytokines and antibodies, and combining it with immune checkpoint inhibitors are critical strategies for enhancing therapeutic efficacy. Future research will focus on precision engineering using CRISPR/Cas9, the development of novel delivery vehicles like nanoparticles and mesenchymal stem cells (MSCs), and biomarker-guided personalized medicine, aiming to provide safer and more effective solutions for refractory cancers. This review synthesizes oHSV advances and analyzes novel delivery and gene-editing strategies.

## 1. Introduction

Oncolytic viruses (OVs) are a class of viruses that can specifically infect and kill tumor cells, possess strong replication capabilities, and can stimulate the body to produce a robust anti-tumor immune response. The concept of using viruses to treat cancer dates back to the early 20th century, with initial clinical observations of improvement in 7 of 22 patients with lymphogranuloma reported in 1912 [1]. In 1949, Moore et al. [2] first discovered in a mouse sarcoma model that the Russian Far East encephalitis virus could selectively infect and kill tumor cells. However, the modern era of oncolytic virotherapy was launched by the advent of genetic engineering. A pivotal moment occurred in 1991 when the first genetically engineered Herpes Simplex Virus type 1 (HSV-1), with its thymidine kinase (TK) gene knocked out, was successfully used to treat mouse glioma [3], marking the beginning of the oHSV field. Among the various OVs being developed, oHSV has emerged as a leading platform for both research and clinical application. Its prominence was cemented in 2015 when the U.S. Food and Drug Administration (FDA) approved Talimogene laherparepvec (T-VEC), an oHSV-based therapy, for the treatment of advanced melanoma [4]. This was followed by the approval of another oHSV, Teserpaturev/G47Δ, in Japan in 2021 for treating malignant glioma, further validating the potential of oHSV in combating solid tumors [5]. In 2024, nanoparticle-enhanced G207 (FA-PEG conjugate) has shown increased tumor targeting in preclinical models [6]. In 2025, MSC-based oHSV delivery demonstrates BBB penetration for brain metastases in clinical trials [7]. These successes have spurred significant investment and research into optimizing oHSV design and application.

This review first explores oHSV’s mechanisms and advantages, then synthesizes preclinical and clinical progress, addresses technical challenges in delivery and administration, and concludes with future directions for personalized therapy.

**Figure 1:**
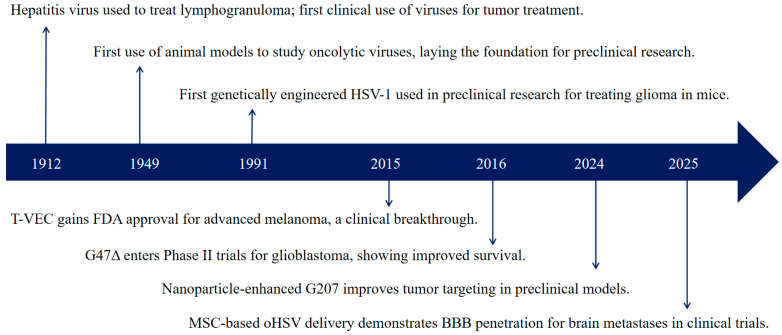
Key milestones in oncolytic herpes simplex virus (oHSV) therapy.

To date, oncolytic virus therapy has become a research hotspot in the field of cancer treatment (Figure 1). Currently, five oncolytic virus drugs have been approved for marketing globally (Table 1).

## 2. Mechanisms and Advantages of oHSV

### 2.1. Core Anti-Tumor Mechanisms

**Figure 2:**
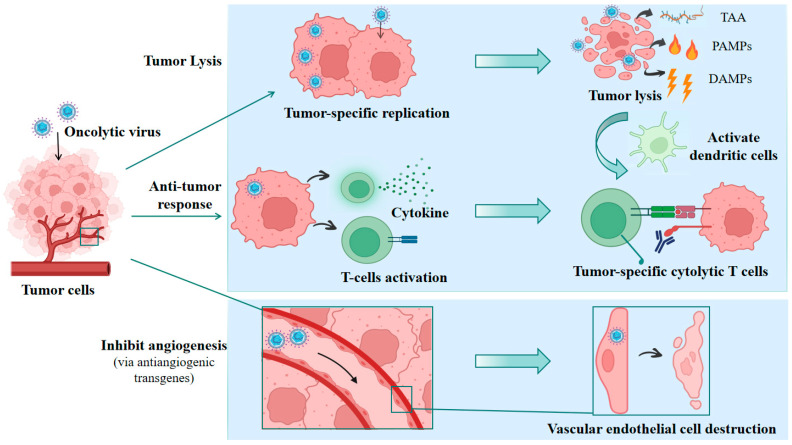
Main mechanisms of tumor cell killing by oncolytic viruses. Oncolysis: Oncolytic viruses replicate and proliferate within tumor cells, leading to cellular lysis. Anti-tumor response: Oncolytic viruses can deliver and release exogenous molecules, activating the host to generate an anti-tumor immune response. Inhibit angiogenesis: Oncolytic viruses can infect tumor-associated vascular endothelial cells, thereby inhibiting tumor angiogenesis. TAA: tumor-associated antigens; PAMPs: Pathogen-Associated Molecular Patterns; DAMPs: Damage-Associated Molecular Patterns.

The anti-tumor mechanisms of oncolytic viruses primarily include the following three (Figure 2): ① Oncolysis: Oncolytic viruses exhibit tumor tropism and can selectively replicate within tumor cells, leading to direct cell lysis. The progeny infectious viruses released after lysis can continue to infect and kill tumor cells [11]. Furthermore, tumor cell lysis releases substances such as tumor-associated antigens (TAAs), Pathogen-Associated Molecular Patterns (PAMPs), and Damage-Associated Molecular Patterns (DAMPs) [12], triggering further immune responses. ② Anti-tumor immune response: Through genetic engineering, one or more exogenous genes (e.g., chemokine genes, immunostimulatory cytokine genes, tumor-associated antigen genes) can be inserted into the oncolytic virus genome. The tumor cells then process and translate these genes into exogenous protein molecules. The expressed chemokines (e.g., CXCL9, CXCL10, CXCL11, etc.) [13] can induce lymphocyte infiltration into tumor tissues. The expressed immunostimulatory factors (e.g., IL-12, IL-15, TNF-α, GM-CSF, etc.) [14] further activate lymphocytes, inducing a strong anti-tumor immune response in the body. The produced tumor-associated antigens [15] can be taken up, processed, and presented by Antigen Presenting Cells (APCs), activating T cells and causing a systemic anti-tumor immune response. This is an important mechanism by which oncolytic virus therapy can exert a killing effect on “distant” tumors. ③ Inhibition of angiogenesis: While not an intrinsic property of wild-type HSV, oHSV can be engineered to inhibit tumor angiogenesis. This is achieved by inserting anti-angiogenic genes, such as those for thrombospondin-1 (TSP-1) [16], angiostatin [17], or endostatin [18,19], into the viral genome. Studies have shown [20] that oncolytic viruses can enter vascular endothelial cells and inhibit intra-tumoral angiogenesis, preventing tumor cells from obtaining the large amounts of nutrients required for growth, thereby achieving growth inhibition.

### 2.2. Unique Advantages of HSV

Based on the type of genetic material, the classification and characteristics of oncolytic viruses are shown in Table 2. Herpes simplex virus (HSV) is a type of herpesvirus belonging to the Alphaherpesvirinae subfamily [21] and is one of the main viruses currently used in the development of oncolytic virus therapies. The structure of HSV consists of a surface envelope, a proteinaceous tegument layer, a nucleocapsid, and a DNA core [22]. Its genome is composed of two unique long (UL) segments and two unique short (US) segments linked together, with terminal repeat sequences. It is classified into two serotypes, HSV-1 and HSV-2 [23]. The HSV-1 genome is 152 kb in length, and the HSV-2 genome is 154 kb in length, with a nucleic acid sequence homology of up to 50% [24].

Compared to other viral vectors, HSV possesses several distinct advantages for oncolytic therapy: ① Large transgene capacity: HSV is an enveloped virus with a complex structure, typically capable of accommodating exogenous genes of approximately 40–50 kb in length, with a maximum capacity of about 100 kb [25]. It is currently the viral vector with the largest capacity, making it possible for HSV to simultaneously carry more and longer immunomodulatory molecule genes. ② High replication efficiency: The virus enters host cells via membrane fusion or endocytosis, releases its DNA into the nucleus, and utilizes the host cell machinery for transcription and replication to complete its life cycle, exhibiting extremely high replication efficiency. ③ Broad tropism: HSV can infect almost all human tumor cells, demonstrating a broad infection spectrum. ④ Blood–brain barrier (BBB) penetration: While the BBB in the body can block most viruses, HSV can attach to and penetrate the BBB, enabling gene delivery to the nervous system. This provides a unique advantage for the treatment of nervous system diseases, especially central nervous system tumors [26]. Compared to other oncolytic viruses (Table 2), HSV holds a prominent position in the development of oncolytic virus therapies due to its unique biological characteristics and modification potential, making it the focus of this review.

**Table 2:** Classification of oncolytic viruses.

Type	Virus Type	Genome Size	Transgene Capacity	Infection Receptor	Cell Entry Mechanism	Replication Site	Advantages
dsDNA	Herpes Simplex Virus (HSV) [27]	150 kb	High	HVEM, nectin1, nectin 2 [28]	Endocytosis; Penetration	Nucleus and Cytoplasm	Large genome, high manipulability
	Adenovirus (Adv) [29]	36 kb	Medium	CAR, CD46	Endocytosis	Nucleus and Cytoplasm	Easy to prepare high-titer virus samples; easy genome manipulation
	Vaccinia Virus (VV) [30]	190 kb	High	GAGs, EFC	Membrane fusion; Endocytosis	Cytoplasm	High virus propagation efficiency; short life cycle; allows insertion of large fragments
dsRNA	Respiratory Enteric Orphan Virus (REO Virus) [31]	16~27 kb	High	JAM-A	Receptor-mediated endocytosis	Cytoplasm	Suitable for intravenous injection; no dose-dependent toxicity
ssDNA	Parvovirus [32]	5 kb	Low	Cyclin A, E2F	Receptor-mediated endocytosis	Nucleus	Tumor tropism, high replication efficiency
(+) ssRNA	Coxsackievirus (CVA) [33]	7.5 kb	Low	CAR, ICAM1, DAF [34]	Micropinocytosis	Cytoplasm	Suitable for intravenous injection
	Seneca Valley Virus (SVV) [35]	7 kb	Low	ANTXR1 [36]	Receptor-mediated endocytosis	Cytoplasm	Non-pathogenic to human
	Poliovirus (PV) [37]	7.5 kb	Medium	CD155	Receptor-mediated endocytosis	Cytoplasm	Infection receptor widely expressed in malignant tumors
(−) ssRNA	Measles Virus (MeV) [38]	16 kb	Low	SLAM, CD46	Membrane fusion	Cytoplasm	Tumor tropism
	Sendai Virus (SeV) [39]	15 kb	Low	Sialic acid	Membrane fusion	Cytoplasm	Tumor tropism; high safety
	Newcastle Disease Virus (NDV) [40]	15 kb	Low	Sialic acid	Endocytosis; pH-independent fusion	Cytoplasm	Non-pathogenic to humans
	Vesicular Stomatitis Virus (VSV) [41]	11 kb	Low	LDLR	Endocytosis	Cytoplasm	Short life cycle; non-pathogenic to humans

dsDNA: double-stranded DNA; dsRNA: double-stranded RNA; ssDNA: single-stranded DNA; (+) ssRNA: positive-sense single-stranded RNA; (−)ssRNA: negative-stranded single-stranded RNA; HVEM: herpesvirus entry mediator; CAR: coxsackie adenovirus receptor; GAGs: glycosaminoglycans; EFC: entry fusion complex; JAM-A: junctional adhesion molecule-A; ICAM1: intercellular adhesion molecule 1; DAF: decay-accelerating factor; ANTXR1: anthrax toxin receptor 1; SLAM: signaling lymphocytic activation molecule; LDLR: low-density lipoprotein receptor.

## 3. Research Progress

### 3.1. Preclinical Advances

As an ideal viral strain for the development of oncolytic virus therapy, recombinant HSV is widely used in cancer immunotherapy. The basic strategy involves modifying or deleting key genes in the HSV genome that affect viral infection and replication to improve targeting and safety and introducing exogenous genes to induce local and systemic immune responses to enhance efficacy. Currently, the main types of genetic modifications include cytokine genes, tumor suppressor genes, anti-angiogenic factor genes, tumor antibody-associated genes, and viral native genes. Below, we will focus on the progress of different genetically modified HSV types in cancer treatment.

#### 3.1.1. Cytokine Genes

Cytokines (CKs) are small-molecule polypeptides or glycoproteins synthesized and secreted by various tissue cells, possessing multiple biological functions such as regulating cell growth and participating in immune responses [42]. In 2015, following the FDA approval of the first genetically engineered HSV (T-VEC) for the treatment of advanced melanoma, a significant amount of research focused on inserting immunostimulatory cytokine genes into the HSV genome (Table 3). These modified HSVs demonstrated good anti-tumor effects and prolonged survival in preclinical animal models. The inserted cytokine genes mainly include GM-CSF [43,44,45,46,47,48,49,50,51], IL-12 [44,45,46,52,53,54,55,56,57], IL-15 [58,59], and OX40L [60] genes. These genes typically activate the local immune microenvironment, promote APC recognition and antigen presentation, stimulate CD4 + T and CD8 + T cell expression, and inhibit regulatory T cell (Treg) function, thereby enhancing the anti-tumor immune response.

Due to immune tolerance in the body, the efficacy of oncolytic viruses administered alone is often suboptimal. Combination therapy is an effective solution to address antiviral immune responses and immune tolerance issues [61]. Currently, treatment regimens often involve combination with immune checkpoint inhibitors (ICIs). Cytokine-modified oHSVs like T-VEC demonstrate robust immune responses, particularly when combined with checkpoint inhibitors, though optimal gene combinations remain under investigation. For example, OncoVEXmGM-CSF, RP1-19, OV-mOX40L, and R-123 have been explored in combination therapy with anti-CTLA-4 or anti-PD-1 antibodies [43,46,47,60]. This approach can alleviate the immunosuppressive tumor microenvironment and prolong the anti-tumor immune response, and the combined application exhibits a “1 + 1 > 2” anti-tumor effect, potentially even overcoming the problem of resistance to monotherapy.

**Table 3:** Preclinical research progress of genetically modified HSV in the last decade—cytokine genes.

Target Gene	Name	Year	Application Method	Tumor Model	ROA	Preclinical Outcome
GM-CSF	OncoVEX ^mGM-CSF^ [43] (HSV-1)	2023	CTLA-4 and PD-1 antibody; 1 × 10^6^ PFU	B16F10	i.t.	Reduced lung metastases, prolonged animal survival.
	RP1-19 [47] (HSV-1)	2020	CTLA-4 and PD-1 antibody; 5 × 10^5^ PFU	TBP-B79	i.t.	Triple combination therapies (PD-1 and CTLA-4 blockade) enhanced antitumor effects.
	OH2 [48,49,50,51] (HSV-2)	2024	1 × 10^6^ CCID_50_/mL	U87, GL261	i.c.	Reduced tumor growth, prolonged animal survival.
		2022	1 × 10^6^, 1 × 10^5^, 1 × 10^4^ CCID_50_/mL	CT26	i.t.	Significant antitumor activity and favorable tolerance
		2022	SIRPα antibody; 2 × 10^6^ PFU	CT26	i.t.	Induction of regional cytokine storm (mainly IL-6).
		2019	2 × 10^7^ CCID_50_/mL	HT-29, CT26	i.t.	OH2 is safe.
OX40L	OV-mOX40L [60] (HSV-1)	2023	IL-6 and PD-1 antibody; 2 × 10^6^ PFU	KPC	i.t.	Improved immunosuppressive microenvironment.
IL-12, IL-15, PD-L1B	VG161 [59,62] (HSV-1)	2020	5 × 10^5^, 5 × 10^6^ PFU	CT26, A20, LS174T	i.t.	Induced robust oncolysis and anti-tumor immune response.
		2023	Paclitaxel; 1 × 10^7^ PFU	EMT-6	i.t.	Reduced breast cancer growth and metastasis.
IL-12, IL-15/IL-15Rα	VG2025 [58] (HSV-1)	2023	1 × 10^6^ PFU	A549	i.t.	Robust antitumor immune response.
IL-12/IL-15/GM-CSF/PD-1 antibody/IL-7, CCL19	oHSV2-IL12, -IL15, -GM-CSF, -PD1v, -IL7 × CCL19 [44] (HSV-2)	2022	1 × 10^7^ PFU	4T1, CT26	i.t.	Combination therapy had better anti-tumor effect.
IL-12, GM-CSF	Δ6/GM/IL12 [45] (HSV-1)	2021	1 × 10^7^ PFU	B16F10	i.t.	The anti-tumor immune response was enhanced.
	R-123 [46] (HSV-1)	2020	PD-1 antibody; 1 × 10^8^ PFU	HER2-LLC1	i.t.	Reduced tumor metastasis.
IL-12	R-115 [52,53] (HSV-1)	2018	2 × 10^9^ PFU	HER2-LLC1	i.p.	Improved immunosuppressive microenvironment.
		2019	2 × 10^6^, 1 × 10^8^ PFU	mHGG^pdgf^-hHER2	i.t.	Reduced tumor growth, improved median survival time.
	M002 [54,55,56,57] (HSV-1)	2017	1 × 10^7^ PFU	SARC	i.t.	Improved immunosuppressive microenvironment.
		2018	1 × 10^7^ PFU	X21415, D456, GBM-12, UAB1016	i.t.	Prolonged animal survival.
		2014	XRT; 1 × 10^7^ PFU	HuH6, G401, SK-NEP-1	i.t.	Reduced tumor growth, prolonged animal survival.
		2013	XRT; 1 × 10^7^ PFU	SK-N-AS, SK-N-BE, Neuro-2a	i.t.	Reduced tumor growth, prolonged animal survival.
	C5252 [63] (HSV-1)	2024	5 × 10^6^ PFU	U87	i.t.	Safe antitumor activity.
CXCL11, IL-12	O-HSV1211 [64] (HSV-1)	2023	1 × 10^7^ PFU	MC38	i.t.	Reduced tumor growth.

XRT: X-ray Radiation Therapy; SIRPα: Signal Regulatory Protein α; ROA: Route of Administration; i.t.: intratumoral injection; i.c.: intracerebral injection; i.p.: intraperitoneal injection.

#### 3.1.2. Tumor Suppressor Genes

Tumor suppressor genes are a class of genes with potential cancer-suppressing effects, playing a negative regulatory role in cell growth, proliferation, and differentiation [65]. Exogenous introduction of PTEN [66,67,68] and P53 [69] genes can enhance the inhibitory effect of HSV on tumor cells (Table 4). HSV expressing PTEN (HSV-P10 [67], oHSV-P10 [68]) has been shown to regulate the PI3K/AKT and IL6/JAK/STAT3 signaling pathways, reduce PD-L1 expression in tumor cells, and decrease tumor immune escape. MH1004 [69], carrying the P53 gene, also showed results of inhibiting tumor growth and prolonging the survival of melanoma mice. Therefore, introducing tumor suppressor genes has also become a feasible option for developing oncolytic virus therapies.

#### 3.1.3. Anti-Angiogenic Factor Genes

Anti-angiogenic factors can affect key signaling pathways that promote angiogenesis, downregulate the expression of vasoactive factors, and inhibit neovascularization [70]. In HSVs modified with thrombospondin-1 (TSP-1) [16], angiostatin [17], and endostatin [18,19] genes (Table 5), treatment resulted in reduced tumor angiogenesis, tumor hypoxia, and necrosis. In 2013, Toshiaki et al. [16] constructed an HSV investigational drug (T-TSP-1) by knocking the TSP-1 gene into the HSV-1 genome. The expressed TSP-1 demonstrated tumor vascular inhibition in the TMK-1 gastric cancer model, indirectly achieving tumor treatment. In 2012, Goodwin et al. [19] constructed an HSV containing another tumor angiogenesis inhibitor, endostatin (HSV-Endo). In a mouse lung metastasis L1C2 model, tumor vascular density was significantly reduced; unfortunately, the expression of endostatin appeared insufficient to achieve complete regression of lung tumors.

#### 3.1.4. Tumor Antibody-Associated Genes

Exogenous introduction of tumor antibody-associated genes also shows unique advantages in developing oncolytic virus therapies (Table 6). Mechanisms of action include utilizing the antibody Fc segment to produce Antibody-Dependent Cell-mediated Cytotoxicity (ADCC) and Antibody-Dependent Cellular Phagocytosis (ADCP) [71], enhancing the immune system’s ability to clear tumors and reducing immunosuppression [72]. In preclinical studies, exogenously introduced anti-Programmed Death-1 (PD-1) antibody genes in glioma [73], ovarian cancer [74], liver cancer [75], colon cancer [76,77], and cutaneous melanoma [77,78] models effectively reduced immunosuppression in the body and enhanced anti-tumor immune responses. For Human Epidermal Growth Factor Receptor 2 (HER2) overexpressing colon cancer [79,80] and lung cancer [81,82,83] models, anti-HER2 antibody gene-recombinant HSV investigational drugs exert anti-tumor effects by inhibiting HER2 homo/heterodimerization, blocking pro-cancer signaling pathways [84], and recruiting immune effector cells (such as NK cells, macrophages) to produce ADCC effects. Anti-Epidermal Growth Factor Receptor (EGFR) antibodies can competitively bind to EGFR, reducing the secretion of immunosuppressive cytokines by tumor cells [85] (such as vascular endothelial growth factor, IL-10). HSV investigational drugs designed and modified accordingly [86,87] have shown good anti-tumor effects in glioblastoma models.

#### 3.1.5. Viral Native Genes

Viral native genes have a significant impact on the safety and efficacy of genetically modified HSV (Table 7). The UL39 gene is involved in encoding ribonucleotide reductase, one of the enzymes necessary for viral replication in non-dividing cells [88]. Knocking out the UL39 gene in the HSV genome can significantly reduce the replication ability of HSV in normal cells, thereby improving safety. The HSV investigational drug G47Δ has a partial deletion of the UL39 gene, which restricts its replication in normal cells while retaining its high replication efficiency in tumor cells [89]. Preclinical studies have shown that G47Δ exhibits good anti-tumor effects in various solid tumor models of the nervous system [90,91,92,93,94,95,96]. Furthermore, combination therapy of G47Δ with chemotherapy drugs (temozolomide [91], axitinib [93]) or ICI drugs (anti-PD-1 and CTLA-4 antibodies [94,95]) has shown even better anti-tumor effects. The ICP34.5 gene is an important virulence factor of HSV, and its expression is closely related to neurotoxicity. Knocking out the ICP34.5 gene can reduce HSV neurotoxicity and minimize damage to the central nervous system. This strategy has been applied in the design of several HSV investigational drugs, such as HSV1716 [97,98,99,100,101,102,103], G207 [104,105], and Virus 16 [106]. The ICP47 gene is involved in regulating the antigen presentation process in host cells. Knocking out this gene can promote the presentation of tumor-associated antigens, enhancing the host immune system’s recognition and attack of tumors [107]. HSV investigational drugs with ICP47 gene knockout, such as G47Δ [90,91,92,93,94,95,96] and Virus 16 [106], have demonstrated more efficient anti-tumor killing effects in various tumor models. In addition to knocking out key genes involved in viral infection and replication, researchers have also focused on genes related to viral particle release and spread. The UL53 gene is involved in regulating the release of viral particles; the gK protein it encodes causes viral particles to accumulate within infected cells, limiting their release [108]. The HSV investigational drug HF10 [109,110,111,112,113,114], which overexpresses the UL53 gene, shows limited release of viral particles after infecting tumor host cells, thereby reducing damage to normal tissues. It has demonstrated good safety and anti-tumor effects in preclinical studies. Currently, regulating genes related to viral particle spread [115] (US3, UL24, etc.) is also receiving considerable attention. Editing these genes aims to optimize HSV spread within tumor tissues while reducing damage to normal tissues.

### 3.2. Clinical Advances

T-VEC’s FDA approval for melanoma and G47Δ’s efficacy in glioblastoma highlight oHSV’s clinical potential, though delivery challenges persist. HSV investigational drugs with different genetic modifications have shown good broad-spectrum anti-tumor effects in preclinical models and are being actively pursued for clinical application. Safety, drug resistance, dosage, and administration route are important factors affecting clinical translation. Currently, clinical studies are underway for various tumors, such as malignant brain tumors, skin or subcutaneous malignant tumors, head and neck cancer, lung cancer, liver cancer, pancreatic cancer, and colorectal cancer.

#### 3.2.1. Malignant Brain Tumors

Glioblastoma, as the most malignant brain tumor [119], is extremely difficult to treat. Among current oncolytic virus therapies (Table 8), G207 has the ICP34.5 gene knocked out to reduce neurotoxicity [120], and G47Δ further deletes the ICP47 gene on this basis to improve viral infection efficiency [27]. Phase I (UMIN000002661) and Phase II (UMIN000015995) clinical studies showed that G47Δ treatment in patients with recurrent glioblastoma resulted in a median survival of 7.3 months, with a one-year survival rate of 38.5% [121,122] and rQNestin34.5v.2 extending overall survival. Other viruses under investigation include rQNestin34.5v.2 [123,124], regulated by the Nestin-1 (Nestin-1) promoter; HSV1716 [125], with ICP34.5 gene deletion; and C134 [126,127], with double-copy ICP34.5 gene deletion and insertion of the Human Cytomegalovirus-TRS1 gene. Clinical data show good safety with intratumoral administration, effective viral replication, and no long-term adverse reactions.

Combination therapy strategies have shown further optimization of efficacy. G207 combined with a single 5Gy radiotherapy (NCT02457845) can increase viral replication and intratumoral spread, enhancing the anti-tumor immune effect [128]. The same regimen is being applied to pediatric malignant gliomas to assess efficacy and safety (NCT04482933). M032, with IL-12 gene knock-in, showed in a Phase I clinical study (NCT02062827) that it could induce increased expression of Interferon-γ (Interferon-γ, IFN-γ) and had a certain therapeutic effect on recurrent malignant glioma [129,130]. Its synergistic mechanism with pembrolizumab is being explored (NCT05084430). These advances highlight the potential of oncolytic viruses in multi-modal combination strategies to improve GBM outcomes.

#### 3.2.2. Skin and Soft Tissue Sarcomas

In clinical studies targeting cutaneous melanoma (Table 9), T-VEC, developed by Amgen, has shown significant efficacy [131]. This investigational drug reduces neurotoxicity by knocking out the ICP34.5 gene of HSV-1 and enhances antigen presentation by knocking out the ICP47 gene while also inserting the GM-CSF gene to boost anti-tumor immune response [132,133,134]. Results from a Phase III clinical trial (NCT00769704) showed that T-VEC monotherapy for advanced melanoma achieved an objective response rate (ORR) of 26%, with a significantly prolonged median survival compared to the control group [135,136]. Notably, 64% of patients experienced regression of distant lesions, suggesting T-VEC can induce systemic anti-tumor immunity [137]. Another clinical study combining T-VEC with ipilimumab (NCT01740297) showed a significantly higher ORR than monotherapy, with disease remission in visceral metastatic sites, indicating a synergistic effect between T-VEC and immune checkpoint inhibitors. [138]. The combination of T-VEC and ipilimumab marks a trend toward synergistic approaches. T-VEC has also demonstrated good therapeutic effects on non-melanoma skin cancers. In 2022, a clinical study (NCT03458117) was initiated to evaluate the efficacy, safety, and tolerability of repeated T-VEC injections in patients with non-melanoma skin cancer. Some reports indicated chronic granulomatous dermatitis at the T-VEC injection site [139], but the nodules spontaneously resolved after stopping injections and did not easily recur [139].

OH2 is one of the few oncolytic virus investigational drugs based on HSV-2. It has the human GM-CSF gene inserted into its genome and has shown durable anti-tumor activity in melanoma and soft tissue sarcoma [140,141], especially for patients who have progressed on anti-PD-1 therapy. Due to the limitations of intratumoral administration, more oHSV investigational drugs are currently in clinical research for skin and other superficial malignant tumors.

#### 3.2.3. Mucosal Epithelial Tumors

Clinical studies indicate that oncolytic HSV has broad-spectrum anti-tumor effects, achieving encouraging results in various mucosal epithelial malignancies such as head and neck cancer, lung cancer, gastric cancer, liver cancer, pancreatic cancer, colorectal cancer, and bladder cancer (Table 10). For example, T-VEC not only exhibits good anti-tumor activity against melanoma, but further studies have found it also has certain therapeutic effects on diseases like head and neck cancer [140], liver cancer [142], and pancreatic cancer [143], enhancing immune-related activity in the body, prolonging patient survival with good safety [144]. Exploration of T-VEC for more indications is ongoing. The efficacy of OH2 monotherapy or in combination with other therapies has been validated in multiple preclinical tumor models [48,49,50,51]. Safety evaluations indicate OH2 is safe and suitable for clinical research [48]. Several clinical studies (NCT03866525, NCT04637698, NCT05232136, and NCT05248789) show that OH2 has broad application prospects in advanced refractory head and neck cancer, gastric cancer, pancreatic cancer, bladder cancer, and other diseases, enhancing the body’s adaptive immune response capability [145] and exhibiting synergistic anti-tumor effects when combined with ICIs [140]. Furthermore, various administration routes have been developed based on the different locations, stages, and progression characteristics of tumors, including intratumoral injection (most common), intravenous injection (NCT05598268, NCT06283303, NCT06200363), nebulization inhalation (NCT06228326), and bladder instillation (NCT06427291, NCT05232136). This demonstrates the characteristics of oncolytic virus therapy, including broad indications and diverse administration routes. While oHSV has shown promise in clinical trials, challenges in delivery and administration limit its efficacy, prompting innovative strategies discussed next.

## 4. Technical Challenges and Solutions

### 4.1. Targeted Delivery

Optimizing the targeting of oncolytic virus therapy has always been central to improving efficacy and safety. Genetic engineering techniques to modify the viral genome can enhance tumor targeting. Key viral genes can be deleted to make replication dependent on tumor-specific pathways. The most common modification is the deletion of the ICP34.5 gene. The viral ICP34.5 protein promotes viral replication by inhibiting the protein kinase R (PKR) and eukaryotic Initiation Factor-2 (eIF2) signaling pathways in normal cells. Knocking out the ICP34.5 gene in HSV blocks its replication in normal cells [146]. In contrast, tumor cells often have an abnormal PKR-eIF2 pathway, allowing HSV lacking the ICP34.5 gene to proliferate only within tumor cells [147], indirectly improving the virus’s tumor cell targeting. Furthermore, surface modification of HSV also affects its targeting. In 2020, Ye et al. [148] covalently modified the HSV drug G207 with a Folate-Poly Ethylene Glycol (FA-PEG) conjugate. This significantly enhanced the targeting of G207 to tumor cells with high folate receptor expression while reducing immunogenicity, demonstrating potential therapeutic value.

To overcome systemic barriers and enhance tumor-specific accumulation, various carriers are being explored. NPs have characteristics such as small size, large surface area, and the ability to cross cellular or tissue barriers [149,150]. They can protect viruses from neutralization and achieve precise delivery [151,152], gradually becoming emerging tools in the biomedical field. In 2024, Totsch et al. [6] studied the pharmacodynamic effects of G207 combined with a self-assembling nanoparticle vaccine (co-delivering antigen peptides and a toll-like receptor 7/8 agonist, called SNAPvax) on the TC-1 model. They found that the combination therapy enhanced the oncolytic effect and induced a more durable T cell immune response. In 2022, Howard et al. [153] combined HSV1716 with nanomagnetic particles (Magnetosomes, MAG) isolated from magnetotactic bacteria. With the aid of an external magnetic field, this approach “navigates” the oncolytic virus, achieving specific tumor targeting while protecting HSV1716 from antibody neutralization. NP biodegradation products can accumulate in cells, potentially causing mutations [154], limiting their role in drug delivery. Therefore, research on the safety and scalability of NPs needs to be strengthened [155] to promote their development in the field of drug therapy.

Biological carriers also show advantages in the targeted delivery of oncolytic viruses. Mesenchymal stem cells (MSCs), as new carriers for targeting tumors and enhancing viral efficacy, are being investigated [156,157,158]. MSCs have been found to migrate to tumor sites via chemotaxis [159,160], possess anti-inflammatory and immunosuppressive properties [161], and induce an immune-tolerant state in the body. This allows loaded oncolytic viruses to evade host immune surveillance and be precisely delivered to tumor tissues [162,163], potentially overcoming the obstacle of local administration only [164]. In 2015, Leoni et al. [165] infected MSCs with HER2-targeted oHSV. Intravenous injection into mice with ovarian cancer lung metastases and breast cancer brain metastases significantly reduced tumor metastasis. Biodistribution assessment showed successful penetration of the BBB, with minimal distribution in normal tissues [164], and activity was maintained after multiple passages [166]. In 2014, Duebgen et al. [167] injected oHSV into hMSCs and found that they could effectively produce progeny oHSV, significantly extending the average lifespan of glioblastoma mice. In 2017, Du et al. [168] studied the therapeutic effect of internal carotid artery (ICA) delivery of MSC-oHSV on brain metastatic melanoma. Fluorescence imaging showed that MSC-oHSV had superior tumor-tracking ability compared to oHSV alone, resulting in a stronger anti-tumor effect. In 2020, Mahasa et al. [169] further confirmed through mathematical modeling that MSC-mediated viral delivery offers better safety and targeting while also demonstrating a synergistic anti-tumor effect. Overall, MSCs have broad application prospects as universal carriers for oncolytic viruses. MSC-based oncolytic virus therapy may also be widely applicable to metastatic lesions in organs such as the liver, colon, and lungs, providing a treatment strategy for metastatic cancer. It also holds promise for overcoming the obstacles of systemic delivery of oncolytic viruses, but issues such as long-term safety [170] and excessive immunosuppression [171] still require systematic research.

### 4.2. Administration Routes

The administration route is key to the effective accumulation of oncolytic viruses at the tumor site and their therapeutic efficacy. Currently, administration routes for oncolytic viruses include systemic administration (intravenous injection) and local administration (intratumoral injection) [172]. Intravenous injection has clear advantages for metastatic tumors, reaching lesions via systemic blood circulation. However, it faces numerous challenges in clinical application: ① Oncolytic viruses entering the bloodstream can be neutralized by pre-existing antiviral antibodies in the serum, leading to direct viral clearance. ② Blood circulates throughout the body, and viral membrane receptors are widely present, making it easy for viruses to infect non-tumor cells, potentially causing damage to normal tissues. ③ Blood can dilute the virus investigational drug, preventing it from enriching at the tumor site and affecting its biodistribution [173]. ④ When tumor lesions are widely distributed and intratumoral blood vessels are abnormally branched and tortuous, the drug can hardly reach every metastatic lesion uniformly [174], affecting treatment efficacy. Therefore, based on the anti-tumor mechanisms of oncolytic viruses and the characteristics of the viruses themselves, most clinical studies of oncolytic virus therapy currently use intratumoral injection. Intratumoral injection allows the drug to directly reach the tumor lesion, which is beneficial for the efficacy and safety of oncolytic virus therapy. However, intratumoral injection may lead to uneven drug distribution within the tumor. Solid tumors have a dense extracellular matrix (ECM) [175], and high permeability of intratumoral blood vessels can lead to high intratumoral pressure [176], hindering viral infiltration. The presence of ECM can impede the spread of oncolytic viruses. Some strategies have been developed, such as pretreatment with enzymes (collagenase or hyaluronidase) or relaxin [177], to promote viral diffusion within tumor tissue. Alternatively, genetic engineering can be used to introduce genes encoding ECM-degrading enzymes into the oncolytic virus genome [178], enabling them to express these enzymes and increase the spread of oncolytic viruses in tumor tissue. Currently, all marketed oncolytic virus drugs are administered intratumorally. Although this allows for precise concentration control at the tumor site, this method is more suitable for superficial tumors, such as melanoma. For metastatic and non-superficial tumors, clinical needs are not yet met. Moreover, the risks associated with intratumoral administration procedures can make repeated dosing difficult [179], causing considerable distress to patients. More convenient administration methods that reduce patient suffering should be investigated to avoid problems associated with the delivery route and mitigate adverse reactions. Developing special administration methods based on tumor characteristics (e.g., nanocarrier delivery [180,181], ultrasound-guided delivery [182]) requires further exploration in future research.

### 4.3. Quality Control

Oncolytic virus products are susceptible to contamination, making the detection of adventitious viruses crucial. Since oncolytic virus products are themselves “live viruses,” distinguishing between the oncolytic virus and adventitious viruses using traditional detection methods like culture-based assays presents significant difficulties. If neutralizing antibodies are used, it is challenging to select antibodies that can effectively neutralize the oncolytic virus. Furthermore, the extensive use of neutralizing antibodies can dilute the test sample, potentially leading to false-negative results. These factors pose challenges to detection methods. oHSV products require stringent quality control to ensure safety and consistency. To better reduce the risk of adventitious virus contamination in oncolytic virus products, strategies can be developed from multiple aspects to ensure quality control, such as purity, potency, and safety testing to ensure the use of appropriate strains [183]. Firstly, cell banks, virus seed lots, and other source materials can all introduce adventitious viruses. Therefore, testing for adventitious viruses is required before using these materials, combined with risk assessment based on their origin, passage history, and raw materials used during bank preparation. For example, T-VEC, which is approved by the FDA, is essentially a live, lytic HSV-1 virus. When samples containing T-VEC are added to indicator cell lines (such as Vero cells) by traditional foreign virus detection methods, the T-VEC self-infection replicates efficiently, resulting in significant cytopathic effect (CPE). This strong effect can “mask” possible low-level exogenous virus contamination, making it impossible to interpret test results and posing a serious product safety and regulatory risk. Comprehensive screening of cell banks and virus seed lots is performed using sensitive methods like polymerase chain reaction (PCR) and gene sequencing to rule out the risk of specific adventitious agent contamination at the source. Secondly, a workaround has been used to develop a high-titer neutralizing antibody specific for oHSV products. It is expected to “neutralize” the foreign virus in the oHSV product so that foreign viral contaminants that are not easily recognized by antibodies are detected in the indicator cells. However, this method has the problem of high development cost, and its validity and reliability need to be considered. To improve the purity and potency of oHSV products, manufacturing processes include robust purification steps like nuclease treatment, ultracentrifugation, and dialysis to remove contaminants [184]. The final product’s infectious potency is quantified using validated methods like plaque assays to ensure consistent dosing [185,186]. Finally, samples are traditionally inoculated into animals such as newborn mice or chicken embryos to test the safety of oHSV products. However, this approach has been widely recognized as scientifically unreliable and unethical. The genetic identity of the oHSV product is confirmed via sequencing to ensure all intended modifications are present and no unintended mutations have occurred.

For an innovative product such as oHSV, the safety testing strategy must not only be scientific and accurate, including uncontrolled viral replication, off-target toxicity, and genetic instability, but also comply with the regulatory requirements of major regulatory agencies around the world [187]. The FDA, which classifies oncolytic viruses as gene therapy or microbial vector products [188], imposes requirements for chemistry, manufacturing, and control through a series of guidelines. Comprehensive characterization of cell banks and virus seeds, strict control of raw materials for production, and comprehensive detection of exogenous agents are emphasized. The European Medicines Agency (EMA) regulates oncolytic viral products through its series of guidelines for advanced therapy medicinal products (ATMPs) [189]. The adoption of a risk-based approach to development and evaluation is emphasized, while covering multiple aspects of quality, nonclinical, and clinical development [190]. The National Medical Products Administration (NMPA) has also issued technical guidelines for oncolytic virus products. Quality risks related to production materials, genetic stability of the virus, and contamination by exogenous factors were clearly pointed out. Enterprises are required to take comprehensive risk control measures to ensure product safety.

## 5. Conclusions and Future Directions

### 5.1. Conclusions

Oncolytic viruses show promising application prospects in tumor therapy, but several technical challenges remain to be addressed. These include issues such as targeting (a key problem in cancer therapy is improving tumor targeting), vector selection (improving viral infection efficiency), and antibody neutralization (viruses entering the body trigger an immune response, producing antibodies that neutralize the virus and reduce efficacy).

Viral vectors carry an inherent risk of pathogenicity, and safety remains the most concerning issue during clinical translation. Although oncolytic viruses are designed to be selective, factors such as non-stringent targeting mechanisms, the influence of the host immune system, or abnormal viral behavior can lead to viruses not accurately infecting tumor cells, resulting in “off-target effects” manifested as local inflammation, tissue damage, and other adverse reactions [191]. In some cases, oncolytic viruses can enter the bloodstream and spread systemically, potentially affecting “distant” tissues and organs; being non-specifically taken up by the lungs, liver, or spleen [192]; and triggering inflammation in non-cancerous sites or autoimmune disease-like reactions [12]. Viral particles in the blood can also activate the complement system, initiating a cascade of protease reactions that lead to the deposition of the membrane attack complex (MAC) [193], causing lysis and destruction of normal cells and a series of adverse reactions. Concurrently, the entry of viruses into the body induces varying degrees of host antiviral immune responses. Currently, this problem cannot be completely resolved at the viral level. Existing approaches often involve combination therapies, suppression of the host immune system, or enhancement of the virus’s ability to induce an anti-tumor response, striving for a balance between antiviral and anti-tumor responses.

Oncolytic viruses are engineered to replicate specifically in tumor cells, lyse them, and release tumor-associated antigens (TAAs), stimulating an adaptive immune response. However, pre-existing immunity to the viral vector and the subsequent production of neutralizing antibodies can affect the intensity and efficiency of the adaptive immune response generated by the body [194]. Therefore, the impact of viral immunogenicity on therapeutic efficacy needs careful consideration. Viral modification processes usually involve the modification or deletion of virulence genes, such as ICP0, ICP34.5, and UL39. The deletion of such genes may affect the immunogenicity of the virus and reduce its replication and spread in tumor cells. Furthermore, HSV therapy typically prioritizes intratumoral injection, and physical barriers like the ECM can limit the biodistribution and spread of the drug [195]. Therefore, combining oncolytic virus therapy with other therapies is crucial, allowing for flexible selection of treatment plans based on the different characteristics of the patient’s tumor and leveraging the respective advantages of different therapies.

The emergence of new technologies is actively promoting oncolytic virus therapy. For instance, nanotechnology offers significant advantages in discovering potential therapeutic targets and predicting biomarkers. Utilizing patient-derived 3D organoids to screen oncolytic virus investigational drugs, employing multi-omics analysis of the tumor microenvironment, and developing oncolytic virus platforms with stronger tumor specificity and higher replication efficiency will facilitate the personalized development of oncolytic virus therapy. The integration of artificial intelligence (AI) algorithms for virus design, prediction of efficacy, and formulation of personalized treatment plans will also be an important direction for HSV therapy. Compared to other viruses, HSV has distinct advantages. Its ability to cross the blood–brain barrier has yet to be further exploited by researchers. It can carry larger exogenous genes than other viral vectors, offering tremendous manipulability. Further advancements in gene-editing technologies like CRISPR/Cas9 will provide more powerful tools for the precise and efficient modification of HSV [64,196,197], holding unparalleled advantages for treating neurological diseases and cancer.

### 5.2. Future Directions

Future core research questions for oHSV will focus on utilizing a new generation of oHSV that can improve tumor heterogeneity and immunosuppressive microenvironment, enhance oncolytic effect, and minimize neurotoxicity. For example, CRISPR/Cas9 and other precision gene-editing tools can be used to create next-generation oHSV. This could involve knocking in/knocking out multiple transgenes, incorporating safety switches, or editing viral glycoproteins to evade neutralizing antibodies. Biomarkers should be identified and validated (e.g., tumor expression of nectin-1, PD-L1 status, or host interferon signature) to select patients most likely to have a response to oHSV therapy, enabling a more personalized approach. Synergistic therapies beyond checkpoint inhibitors, including with CAR-T cell therapy, targeted small molecule inhibitors, and radiotherapy, can overcome drug resistance and enhance efficacy. Artificial intelligence learning algorithms can be employed to design novel virus backbones, predict therapeutic efficacy, and formulate personalized treatment regimens based on patient-specific tumor data.

## Figures and Tables

**Table 1:** Overview of globally marketed oncolytic virus drugs.

Drug Name	Year of Approval	Viral Vector	Therapeutic Target	Indications	Company
Rigvir [8]	2004	ECHO virus	/	Melanoma, colorectal cancer	Latima, Riga, Latvia
H101 (Oncorine) [9]	2005	Human adenovirus-5	E1B-55kDa, E3-19kDa	Head and neck cancer	Sunway Biotech, Shanghai, China
T-VEC (Lmlygic) [4]	2015	Herpes simplex virus-1	Deletion of ICP34.5, ICP47; insertion of hGM-CSF	Advanced melanoma	Amgen, Thousand Oaks, CA, USA
G47Δ (Delytact) [5]	2021	Herpes simplex virus-1	Deletion of ICP34.5, ICP47; insertion of LacZ	Malignant glioma, primary brain tumor	Daiichi Sankyo, Tokyo, Japan
Adstiladrin [10]	2022	Non-replicating adenovirus	Insertion of IFN-α2b	Non-muscle invasive bladder cancer	Ferring, Parsippany, NJ, USA

ECHO virus: Enteric Cytopathic Human Orphan virus.

**Table 4:** Preclinical research progress of genetically modified HSV in the last decade—tumor suppressor genes.

Target Gene	Name	Year	Application Method	Tumor Model	ROA	Preclinical Outcome
PTEN	HSV-P10 [67] (HSV-1)	2018	1 × 10^5^ PFU	DB7 U87ΔEGFR	i.t.	Overcame tumor immune escape.
	oHSV-P10 [68] (HSV-1)	2023	2 × 10^5^ PFU	GBM-12 005 GSCs	i.t.	Reduced tumor growth.
	PTEN-VP22 [66] (HSV-1)	2016	100 μg	Eca-109	i.t.	Increased the antitumor activity of PTEN.
P53	MH1004 [69] (HSV-1)	2016	2 × 10^6^ PFU	B16-F10	i.t.	Reduced tumor growth, prolonged animal survival.

PTEN: phosphatase and tensin homolog deleted on chromosome ten; i.t.: intratumoral injection.

**Table 5:** Preclinical research progress of genetically modified HSV in the last decade—anti-angiogenic factor genes.

Target Gene	Name	Year	Application Method	Tumor Model	ROA	Preclinical Outcome
TSP-1	T-TSP-1 [16] (HSV-1)	2013	1 × 10^7^ PFU	TMK-1, MKN1	i.t.	Reduced tumor angiogenesis.
Angiostatin	G47Δ-mAngio [17] (HSV-1)	2013	G47Δ-mIL12; 1 × 10^6^ PFU	GSCs, U87	i.t.	Reduced tumor growth.
Endostatin	HSV-Endo [19] (HSV-1)	2012	1 × 10^7^ PFU	L1C2	i.t.	Reduced vascular density, incomplete regression.
	VAE [18] (HSV-1)	2014	5 × 10^4^ PFU	GBM-SCs	i.t.	Reduced tumor growth.

TSP-1: thrombospondin-1; i.t.: intratumoral injection.

**Table 6:** Preclinical research progress of genetically modified HSV in the last decade—tumor antibody-associated genes.

Target Gene	Name [77,78]	Year	Application Method	Tumor Model	ROA	Preclinical Outcome
PD-1 antibody	NG34scFvPD-1 [73,74] (HSV-1)	2019	1.5 × 10^6^ PFU	GL261, CT2A	i.t.	Induced durable antitumor response.
		2023	PI3K inhibitor; 1 × 10^6^ PFU	ID8	i.t.	Reduced tumor growth, prolonged animal survival.
	YST-OVH [75] (HSV-1)	2022	1 × 10^7^ PFU	Hepa1-6	i.t.	Antitumor immunity and safe.
	HSV-aPD-1 [77] (HSV-1)	2021	1 × 10^7^ PFU	MC38, B16-F10	i.t.	Reduced tumor growth.
	VT1903M [76] (HSV-1)	2022	1 × 10^7^ PFU	CT26	i.t.	Reduced tumor growth.
	oHSV2-aPD1 [78] (HSV-2)	2019	2 × 10^5^ PFU	B16R	i.t.	Induced durable antitumor response.
HER2 antibody	VG22401 [79] (HSV-1)	2023	1 × 10^7^ PFU	CT26	i.t.	Enhanced antitumor immunity and efficacy.
	R337 [80] (HSV-1)	2021	1 × 10^7^, 1.5 × 10^7^, 5 × 10^7^ PFU	CT26-HER2	i.t.	Enhanced antitumor immunity.
	R-335 [81] (HSV-1)	2021	1 × 10^8^ PFU	HER2-LLC1	i.t.	Improved immunosuppressive microenvironment.
	R-LM113 [82] (HSV-1)	2020	PD-1 antibody; 1 × 10^8^ PFU	HER2-LLC1	i.t.	Sting provides fundamental contributions to immunotherapeutic efficacy.
	R87 [83] (HSV-1)	2018	1 × 10^8^ PFU	HER2-LLC1	i.t.	Targeted HER2^+^ cancer cells.
EGFR antibody	OV-Cmab-mCCL5 [86] (HSV-1)	2022	2 × 10^5^ PFU	CT2A-hEGFR	i.t.	Reduced tumor growth, prolonged animal survival.
	R-613 [87] (HSV-1)	2021	1 × 10^9^ PFU	GBM	i.t.	Increased animal median survival time.

Sting: stimulator of interferon genes; i.t.: intratumoral injection.

**Table 7:** Preclinical research progress of genetically modified HSV in the last decade—viral native genes.

Target Gene	Mechanism of Action	Name	Year	Application Method	Tumor Model	ROA	Preclinical Outcome
gD (US6)	Binds to HVEM/nectin-1, promotes membrane fusion	R-LM249 [116] (HSV-1)	2013	2 × 10^7^, 1 × 10^8^ PFU	SK-OV-3, MDA-MB-453	i.p.	Reduced tumor growth, 95% reduction of neoplastic nodules.
ICP34.5 (RL1)	Reduces neurotoxicity; enhances tumor infection specificity	HSV1716 [97,98,99,100,101,102,103] (HSV-1)	2014	2 × 10^6^, 1 × 10^6^ PFU	HuH7, HepG2	i.t. i.v.	Reduced tumor growth, prolonged animal survival.
			2017	5 × 10^6^ PFU	DIPG	i.t.	Inhibited brain tumor migration and invasion.
			2017	A8301; 1 × 10^8^ PFU	RMS	i.t.	Prolonged animal survival, some complete responses.
			2017	Alisertib; 1 × 10^7^ PFU	S462TY, SK-N-AS	i.t.	Reduced tumor growth, prolonged animal survival.
			2021	1 × 10^6^ PFU	PyMT-TS1, 4T1, E0771	i.v.	Reduced tumor growth, prolonged animal survival.
			2023	Bortezomib; 1 × 10^6^ PFU	JJN-3, 5TGM1	i.v.	Lower tumor burden rates, prevented myeloma cell regrowth.
			2022	BRAFi; 5 × 10^5^ PFU	4434, Mel888	i.t.	Enhanced survival, but cannot fully control tumors.
		G207 [104,105] (HSV-1)	2012	1 × 10^7^ PFU	HT29, PLC5	i.t.	Provided potential targets to overcome resistance.
			2016	1 × 10^7^ PFU	D425, D341	i.t.	Pediatric medulloblastoma may be an excellent target.
ICP34.5, ICP47 (US12); GALV-GP-R^−^	Enhances viral oncolytic effect, increases immunogenic cell death	Virus 16 [106] (HSV-1)	2019	CTLA-4 antibody; 5 × 10^6^ PFU	A20, A549, MDA-MB-231	i.t.	Reduced tumor growth.
ICP34.5, ICP47 (US12)		VT09X [117] (HSV-1)	2022	Pembrolizumab; 1 × 10^7^ PFU	B16-F10	i.t.	Antitumor immune response, prolonged animal survival.
ICP6 (UL39), ICP47, US11	Promotes viral DNA synthesis; affects MHC-I molecule expression	G47Δ [90,91,92,93,94,95,96] (HSV-1)	2014	3 × 10^6^ PFU	MPNST, S462	i.t.	Reduced tumor growth, prolonged animal survival.
			2017	PD-1 and CTLA-4 antibody; 5 × 10^5^ PFU	005 GSCs, CT-2A	i.t.	Prolonged animal survival.
			2020	O6-BG, TMZ; 5 × 10^5^ PFU	005 GSCs	i.t.	Prolonged animal survival.
			2020	2 × 10^6^ PFU	4T1	i.t.	Reduced tumor burden and metastasis.
			2018	PD-1 and CTLA-4 antibody; 5 × 10^5^ PFU	005 GSCs	i.t.	Antitumor immune response.
			2018	Axitinib; 2.5 × 10^5^ PFU	005 GSCs, MGG123	i.t.	Prolonged animal survival.
			2024	5 × 10^5^ PFU	005 GSCs, CT-2A, GL261	i.t.	Stimulated antitumor immunity, prolonged median survival.
VP16 (UL48)	Initiates immediate-early gene transcription	KM100 [118] (HSV-1)	2016	2 × 10^7^ PFU	TUBO	i.t.	Prolonged animal survival.
gK (UL53), ICP27 (UL54)	Promotes membrane fusion; affects mRNA splicing	HF10 [109,110,111,112,113,114] (HSV-1)	2014	Erlotinib; 1 × 10^5^ PFU	BxPC3, PANC-1	i.t.	Combination therapy is more effective.
			2021	1 × 10^7^ PFU	NMOC1	i.t.	Prolonged animal survival.
			2017	1 × 10^7^ PFU	MC26	i.t.	Inhibited tumor metastasis.
			2017	DTIC; 1 × 10^7^ PFU	clone M3	i.t.	Induced anti-tumor immune response and prolonged survival.
			2019	Cetuximab; 5 × 10^6^ PFU	HT-29	i.t.	Antitumor immune response, suppressed angiogenesis.
			2020	1.5 × 10^6^ PFU	FaDu, SCC-VII	i.t.	Reduced tumor growth, prolonged animal survival.

GALV-GP-R^−^: envelope glycoprotein of gibbon ape leukemia virus; BRAFi: BRAF^V600E^ mutant-specific inhibitor; DTIC: dacarbazine; O6-BG: O6-Benzylguanine; TMZ: Temozolomide; i.p.: intraperitoneal injection; i.t.: intratumoral injection; i.v.: intravenous injection.

**Table 8:** Overview of clinical research of genetically modified HSV in the last decade—neuroectodermal tumors.

Name	Target	Indications	Combination Therapy	Phase/Status	ROA	Year	Clinical Trial No.	Clinical Outcome
HSV1716	ICP34.5	Malignant Glioma	/	Phase I/Terminated	i.t.	2013	NCT02031965	Data not reported.
C134	ICP34.5; HCMV-TRS1	Recurrent Glioblastoma	/	Phase Ib/Active, not recruiting	i.t.	2024	NCT06193174	Clinical studies are ongoing.
			/	Phase I/Active, not recruiting	i.t.	2019	NCT03657576	
		Malignant Glioma	/	Phase II/Active, not recruiting	i.t.	2024	NCT06614855	
G47Δ	ICP34.5, ICP47; LacZ	Malignant Glioma	/	Phase I-II/Completed	i.t.	2019	UMIN000002661	Median OS 23.3 months, 1-year survival rate 92.3%.
			/	Phase II/Completed	i.t.	2020	UMIN000015995	
rQNestin34.5v.2	ICP34.5, UL39	Malignant Glioma	/	Phase I/Recruiting	i.t.	2017	NCT03152318	Clinical studies are ongoing.
G207	ICP34.5; LacZ	Malignant Glioma	5Gy radiotherapy	Phase II/Recruiting	i.t.	2024	NCT04482933	
		Recurrent Brain Tumor	5Gy radiotherapy	Phase I/Recruiting	i.t.	2019	NCT03911388	
				Phase I/Completed	i.t.	2020	NCT02457845	Median OS 23.3 months.
ON-01	TK, RR, UNG	Malignant Glioma	/	Phase I-II/Completed	i.t.	2022	NCT06562621	Data not reported.
MVR-C5252	ICP34.5; IL-12, PD-1	Malignant Glioma	/	Phase I/Recruiting	i.t.	2024	NCT06126744	Clinical studies are ongoing.
M032	ICP34.5; IL-12	Recurrent Malignant Glioma	/	Phase I/Active, not recruiting	i.t.	2022	NCT02062827	Data not reported.
			Pembrolizumab	Phase I-II/Recruiting	i.t.	2022	NCT05084430	Clinical studies are ongoing.
OH2	GM-CSF	Recurrent Glioblastoma	/	Phase I-II/Recruiting	i.t.	2021	NCT05235074	

HCMV-TRS1: Human Cytomegalovirus-TRS1; LacZ: β-galactosidase gene; TK: thymidine kinase; RR: ribonucleotide reductase; UNG: uracil DNA glycosylase; OS: overall survival; i.t.: intratumoral injection.

**Table 9:** Overview of clinical research of genetically modified HSV in the last decade—skin and soft tissue sarcomas.

Name	Target	Indications	Combination Therapy	Phase/Status	ROA	Year	Clinical Trial No.	Clinical Outcome
T-VEC	GM-CSF	Melanoma	/	Phase III/Completed	i.t.	2014	NCT00769704	ORR 31.5%, median OS 23.3 months, DRR 19%.
			EBRT	Phase II/Completed	i.t.	2016	NCT02819843	0% grade 3 AEs.
			Ipilimumab	Phase II/Completed	i.t.	2021	NCT01740297	ORR 35.7%, median PFS 13.5 months.
			Nivolumab	Phase II/Completed	i.t.	2020	NCT04330430	Pathologic CR 45%.
			Pembrolizumab	Phase II/Recruiting	i.t.	2019	NCT03842943	Clinical studies are ongoing.
		Advanced Soft Tissue Sarcoma	EBRT	Phase I/Recruiting	i.t.	2024	NCT06660810	
			Nivolumab	Phase II/Recruiting	i.t.	2019	NCT03886311	
		Non-melanoma Skin Cancer	/	Phase I/Completed	i.t.	2022	NCT03458117	Data not reported.
ONCR-177	IL-12, CCL4, FLT3LG, PD-1 and CTLA-4 antibodies	Skin/Subcutaneous Malignancies	Pembrolizumab	Phase I/Terminated	i.t.	2020	NCT04348916	Data not reported.
OrienX010	GM-CSF	Malignant Melanoma	/	Phase I/Unknown	i.t.	2016	NCT03048253	Data not reported.
		Melanoma	/	Phase I/Completed	i.t.	2012	NCT01935453	
T3011	IL-12, PD-1 antibody	Non-melanoma Skin Cancer, Sarcoma	/	Phase I-II/Unknown	i.t.	2020	NCT05602792	Results unknown.
RP1	GM-CSF, GALV-GP R^-^	Melanoma	/	Phase I/Recruiting	i.t.	2024	NCT06216938	Clinical studies are ongoing.
		Squamous Cell Carcinoma	/	Phase I-II/Recruiting	i.t.	2023	NCT05858229	
		Advanced Skin Malignancies	/	Phase I-II/Recruiting	i.t.	2020	NCT04349436	
		Non-melanoma Skin Cancer	Nivolumab	Phase II/Recruiting	i.t.	2017	NCT03767348	
RP2	GM-CSF, GALV-GP R^-^, CTLA-4 antibody	Metastatic Uveal Melanoma	Nivolumab	Phase II-III/Recruiting	i.t.	2024	NCT06581406	Clinical studies are ongoing.
HF10	UL43, UL49.5, UL55, UL56, LAT	Melanoma	Nivolumab	Phase II/Completed	i.t.	2018	NCT03259425	ORR 83.3%, 14.3% grade 3 AEs.
			Ipilimumab	Phase II/Completed	i.t.	2018	NCT03153085	Data not reported.
						2016	NCT02272855	
			/	Phase I/Completed	i.t.	2015	NCT01017185	
OH2	GM-CSF	Melanoma	Pembrolizumab	Phase I-II/Recruiting	i.t.	2018	NCT04386967	Clinical studies are ongoing.
			PD-1 antibody HX008	Phase I-II/Recruiting	i.t.	2020	NCT04616443	
			/	Phase III/Recruiting	i.t.	2023	NCT05868707	
		Soft Tissue Sarcoma	PD-1 antibody HX008	Phase I-II/Recruiting	i.t.	2019	NCT03866525	
R130	CD3 scFv, CD86, PD-1, HSV2-US11	Melanoma	/	Phase I/Recruiting	i.t.	2023	NCT05961111 NCT06171282	Clinical studies are ongoing.
		Advanced Bone/Soft Tissue Tumors	/	Phase I/Recruiting	i.t.	2023		
							NCT05851456	
		Sarcoma	/	Phase I/Recruiting	i.t.	2023	NCT05860374	
KB707	IL-2, IL-12	Melanoma	/	Phase I-II/Recruiting	i.t.	2023	NCT05970497	
HSV1716	ICP34.5	Sarcoma	/	Phase I/Completed	i.t.	2018	NCT00931931	Data not reported.

EBRT: External Beam Radiation Therapy; FLT3LG: FMS-like Tyrosine Kinase-3 Ligand; LAT: Latency-associated Transcripts; ORR: objective response rate; OS: overall survival; DRR: durable response rate; AE: adverse event rates; PFS: progression-free survival; CR: complete response; i.t.: intratumoral injection.

**Table 10:** Overview of clinical research of genetically modified HSV in the last decade—mucosal epithelial tumors.

Indications	Name	Target	Combination Therapy	Phase/Status	ROA	Year	Clinical Trial No.	Clinical Outcome
**Respiratory System**								
Head and Neck Cancer	T3011	IL-12, PD-1 antibody	/	Phase I–II/Unknown	i.t.	2020	NCT05602792	Results unknown.
	T-VEC	GM-CSF	Pembrolizumab	Phase I/Completed	i.t.	2017	NCT02626000	Median PFS 3.0 months, OS 5.8 months.
	HF10	UL43, UL49.5, UL55, UL56, LAT	/	Phase I/Completed	i.t.	2015	NCT01017185	Data not reported.
	OH2	GM-CSF	PD-1 antibody HX008	Phase I–II/Recruiting	i.t.	2019	NCT03866525	Clinical studies are ongoing.
	R130	CD3 scFv, CD86, PD-1, HSV2-US11	**/**	Phase I/Recruiting	i.t.	2023	NCT05961111	
			**/**	Phase I/Unknown	i.t.	2023	NCT05886075	Results unknown.
			/	Phase I/Recruiting	i.t.	2023	NCT05830240	Clinical studies are ongoing.
Lung Cancer	OrienX010	GM-CSF	/	Phase I/Completed	i.t.	2012	NCT01935453	Data not reported.
	T3011	IL-12, PD-1 antibody	/	Phase I–II/Unknown	i.v.	2022	NCT05598268	Results unknown.
	RP2	GM-CSF, GALV-GP R^-^, CTLA-4 antibody	Nivolumab	Phase I/Recruiting	i.t.	2019	NCT04336241	Clinical studies are ongoing.
	R130	CD3 scFv, CD86, PD-1, HSV2-US11	/	Phase I/Recruiting	i.t.	2023	NCT05961111	
			/	Phase I/Unknown	i.t.	2023	NCT05886075	Results unknown.
	KB707	IL-2, IL-12	/	Phase I–II/Recruiting	nebulization	2024	NCT06228326	Clinical studies are ongoing.
Pleural Mesothelioma	HSV1716	ICP34.5	/	Phase I–II/Completed	i.p.	2016	NCT01721018	Data not reported.
**Digestive System**								
Gastric Cancer	VG161	IL-12, IL-15, PD-L1B	Nivolumab	Phase I–II/Recruiting	i.t.	2022	NCT06008925	Clinical studies are ongoing.
	OH2	GM-CSF	PD-1 antibody HX008	Phase I–II/Recruiting	i.t.	2019	NCT03866525	
Liver Cancer	OrienX010	GM-CSF	/	Phase I/Completed	i.t.	2012	NCT01935453	Data not reported.
	T3011	IL-12 and PD-1 antibody	/	Phase I–II/Unknown	i.v.	2022	NCT05598268	Results unknown.
	T-VEC	GM-CSF	/	Phase I/Completed	i.t.	2018	NCT03256344	ORR 10%, PFS 5.4 months, OS 19.2 months.
	RP2	GM-CSF, GALV-GP R-, CTLA-4 antibody	Nivolumab	Phase I/Recruiting	i.t.	2019	NCT04336241	Clinical studies are ongoing.
			Atezolizumab and Bevacizumab	Phase II/Recruiting	i.t.	2024	NCT05733598	
	VG161	IL-12, IL-15, PD-L1B	Camrelizumab	Phase I-II/Not yet recruiting	i.t.	2023	NCT06124001	
			/	Phase I/Recruiting	i.t.	2021	NCT04806464	
	R130	CD3 scFv, CD86, PD-1, HSV2-US11	/	Phase I/Recruiting	i.t.	2023	NCT05860374	
Pancreatic Cancer	OrienX010	GM-CSF	/	Phase I/Completed	i.t.	2012	NCT01935453	Data not reported.
	HF10	UL43, UL49.5, UL55, UL56, LAT	Gemcitabine + Paclitaxel	Phase I/Active, not recruiting	i.t.	2020	NCT03252808	Clinical studies are ongoing.
			Erlotinib + Gemcitabine	Phase I/Completed	i.t.	2018	UMIN000010150	Median PFS 6.3 months, median OS 15.5 months.
	T-VEC	GM-CSF	/	Phase I/Completed	i.t.	2017	NCT03086642	Median OS 7.8 months.
	VG161	IL-12, IL-15, PD-L1B	Nivolumab	Phase I-II/Recruiting	i.t.	2022	NCT05162118	Clinical studies are ongoing.
	OH2	GM-CSF	/	Phase I-II/Terminated	i.t.	2021	NCT04637698	Results unknown.
Colorectal Cancer	ONCR-177	IL-12, CCL4, FLT3LG, PD-1 and CTLA-4 antibody	Pembrolizumab	Phase I/Terminated	i.t.	2020	NCT04348916	Results unknown.
	T3011	IL-12 and PD-1 antibody	Toripalimab + Regorafenib	Phase I/Recruiting	i.v.	2024	NCT06283303	Clinical studies are ongoing.
			Regorafenib	Phase I/Recruiting	i.v.	2023	NCT06200363	
	R130	CD3 scFv, CD86, PD-1, HSV2-US11	/	Phase I/Recruiting	i.t.	2023	NCT05860374	
**Urogenital System**								
Bladder Cancer	T3011	IL-12 and PD-1 antibody	/	Phase I/Recruiting	bid.	2023	NCT06427291	Clinical studies are ongoing.
	OH2	GM-CSF	/	Phase I-II/Recruiting	bid.	2022	NCT05232136	
			/	Phase II/Recruiting	i.t.	2022	NCT05248789	
Ovarian Cancer	R130	CD3 scFv, CD86, PD-1, HSV2-US11	/	Phase I/Recruiting	i.t. or i.v.	2022	NCT05801783	
Cervical Cancer			/	Phase I/Recruiting	i.t. or i.v.	2023	NCT05812677	
	BS-006	CD3 and PD-L1 antibod	/	Phase I/Recruiting	i.t.	2022	NCT05393440	

i.t.: intratumoral injection; i.v.: intravenous injection; i.p.: intraperitoneal injection; bid.: bladder instillation drip; PFS: progression-free survival; OS: overall survival; ORR: objective response rate.
